# Hypomelanosis of Ito with a trisomy 2 mosaicism: a case report

**DOI:** 10.1186/1752-1947-8-333

**Published:** 2014-10-09

**Authors:** Giovanni Ponti, Giovanni Pellacani, Aldo Tomasi, Antonio Percesepe, Carmelo Guarneri, Azzurra Guerra, Victor Desmond Mandel, Elif Kisla, Piril Cevikel, Claudia Neri, Cristina Menozzi, Stefania Seidenari

**Affiliations:** 1 Department of Clinical and Diagnostic Medicine and Public Health, University of Modena and Reggio Emilia, via del Pozzo 71, 41124 Modena, Italy; 2 Department of Mother & Child, Unit of Medical Genetics, University of Modena and Reggio Emilia, via del Pozzo 71, 41124 Modena, Italy; 3 Department of Dermatology, University of Modena and Reggio Emilia, via del Pozzo 71, 41124 Modena, Italy; 4 Department of Pediatrics, University of Modena and Reggio Emilia, via del Pozzo 71, 41124 Modena, Italy; 5 Faculty of Medicine, Akdeniz University, Dumlupinar Boulevard, 07100 Antalya, Turkey

**Keywords:** Hypomelanosis of Ito, Trisomy 2 mosaicism, Familial hypomelanosis of Ito, Neurocutaneous disorder, Blaschko lines

## Abstract

**Introduction:**

Hypomelanosis of Ito is a rare neurocutaneous disorder, characterized by streaks and swirls of hypopigmentation following the lines of Blaschko that may be associated to systemic abnormalities involving the central nervous system and musculoskeletal system. Despite the preponderance of reported sporadic hypomelanosis of Ito, few reports of familial hypomelanosis of Ito have been described.

**Case presentation:**

A 6-month-old Caucasian girl presented with unilateral areas of hypomelanosis distributed on the left half of her body and her father presented with similar mosaic hypopigmented lesions on his upper chest. Whereas both blood karyotypes obtained from peripheral lymphocyte cultures were normal, a 16% trisomy 2 mosaicism was found in cultured skinfibroblasts derived from a hypopigmented skin area of her father.

**Conclusions:**

Familial cases of hypomelanosis of Ito are very rare and can occur in patients without systemic involvement. Hypomelanosis of Ito constitutes a non-specific diagnostic definition including different clinical entities with a wide phenotypic variability, either sporadic or familial. Unfortunately, a large number of cases remain misdiagnosed due to both diagnostic challenges and controversial issues on cutaneous biopsies in the pediatric population.

## Introduction

Hypomelanosis of Ito (HMI) is a neurocutaneous phenotype characterized by hypopigmented anomalies along the Blaschko lines, with systemic abnormalities involving the central nervous and muscle-skeletal systems. It was firstly described as incontinentia pigmenti achromians in 1952 [1]. Because of its complex diagnosis the precise prevalence is difficult to estimate, but it has been calculated that HMI is present in 1 in 3,000 to 1 in 10,000 children [2]. Both sexes are affected with an approximately 2:1 female preponderance [3]. The clinical manifestations vary from small hypopigmented areas to hemisomatic large hypopigmented whorls. The streaks can be unilateral or bilateral and, generally, they follow the lines of Blaschko. Lesions appear within the first year of life in about three quarter of the patients. The different pigmented skin areas correspond to the distribution of the two distinct cell clones with different pigment potential in single individuals. The extra-cutaneous manifestations (such as scoliosis, vertebral anomalies and craniofacial malformations) involve the central nervous system and muscle-skeletal system in 33 to 94% of the cases, respectively [4]. However, cardiac, genitourinary and ophthalmic anomalies have been described. These extra-cutaneous manifestations probably do not reflect the variability of one single disorder, but can be due to the presence of different genetic defects. Usually HMI is considered a sporadic disorder, but dominant and recessive (including X-linked) inheritance has also been reported [1-3]. HMI can be due to gametic half chromatid or somatic mutations, or to chromosomal mosaicisms [5], which have been demonstrated in some of the affected patients by means of skin biopsies. Moreover, an association between HMI and ring chromosome 20, trisomy of chromosome 13, and other cytogenetic abnormalities have been described [2]. In this paper we present the clinical and cytogenetic characterization of a familial case of HMI (father and daughter), and we analyze all the familial cases of HMI reported in scientific literature. The criteria set forth by Ruiz-Maldonado *et al.* for the diagnosis of HMI were used [6]. Chromosomes were processed by standard GTG- and/or QFQ-banding techniques and analyzed at a resolution of 400 bands for a haploid set [International System for Human Cytogenetic Nomenclature (ISCN), 2013] for both the peripheral blood and fibroblasts from skin biopsy. A minimum of 100 metaphase spreads from two separate cultures for the peripheral blood, and six for the skin biopsy, were examined.

## Case presentation

**Figure 1:**
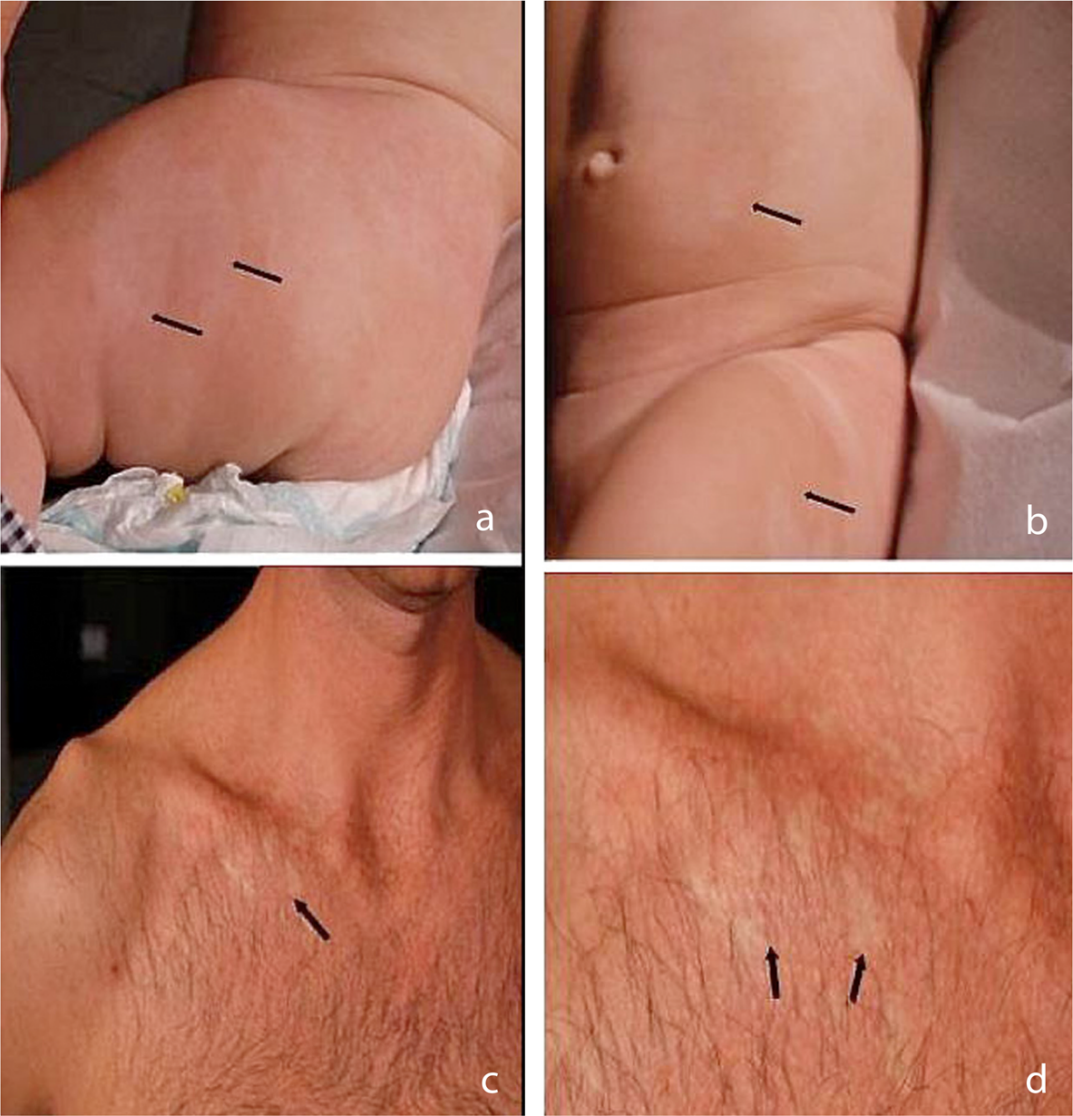
**Clinical features of two probands. (a and b)** right hemisomatic hypopigmented streaks of the trunk and of one leg in our patient; **(c and d)** small hypopigmented areas on the upper chest in her father.

**Figure 2:**
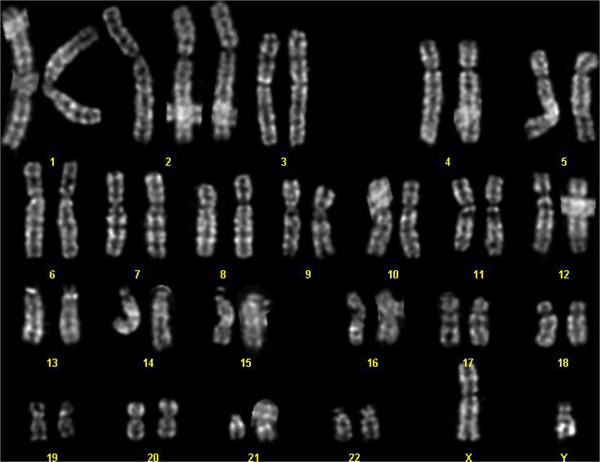
Karyotypic analysis from fibroblast cultures obtained from a skin biopsy of the hypopigmented area showing trisomy 2.

A 6-month-old Caucasian girl presented to our department for the evaluation of right hemisomatic hypopigmented streaks of the trunk and of one leg noted at 3-months-old after sun exposure. At no time were vesicobullous, lichenoid, or verrucous lesions observed and on clinical examination she did not show any other extra-cutaneous manifestations. Her growth and development since birth were within normal limits. Her physical examination was unremarkable, except for the presence of hypopigmented areas on the right leg and on the trunk and back in a pattern which did not cross the midline (Figure 1). Regarding the family history and examination of family members, similar hypomelanotic lesions had been present in her father since birth, but no other peculiar signs or symptoms were present in her genealogic tree. Her mother, 39-years-old at the time of delivery, was in excellent health. Moreover, there was no family history of congenital nervous or systemic abnormalities. The karyotypic analysis of the peripheral blood cultures of our patient and her father did not reveal any chromosomal anomaly. The karyotypic analysis from fibroblast cultures obtained from a skin biopsy of the hypopigmented area of the father of our patient showed the presence of a trisomy 2 cell line in a 16% mosaic with a normal cell line (karyotype: mos47,XY,+2(15)/46,XY(90); Figure 2). Her parents decided not to authorize the excisional biopsy on their daughter and the fibroblast karyotypic analysis could not be performed.

## Discussion

To the best of our knowledge only 15 reports of familial HMI have been described in the current literature (Table 1). HMI may present in both sexes and dominant, recessive and X-linked inheritances have been reported [1-3] as chromosomal aberrations of this rare disorder. HMI may also be transmitted from parent to child with variable presentation and expressivity (Table 1). In the HMI family described by Sacrez *et al*. and Grosshans *et al*. [7,8], the mother and her three daughters were all affected. The daughters showed clinical and histopathological cutaneous changes typical of HMI, marked psychomotor retardation, strabismus, hypodontia and skeletal dysplasia. The mother had only non-specific hypopigmented areas. The inheritance in this family with four female individuals suggested an X-linked dominant trait. Cram and Fukuyama described HMI in a child, his mother and his maternal grandfather [9]. Patrizi *et al*. [10] reported two siblings, a boy and a girl, with typical HMI and neurological symptoms. The mother had typical pigmentary abnormalities without neurological defects [10]. Vormittag *et al*. [3] reported a family with an affected mother and daughter that was probably consistent with HMI. The mother showed hypopigmentation, eye anomalies, epileptic seizures and skeletal abnormalities. Her daughter displayed hypopigmentation following the lines of Blaschko and eye anomalies [3]. A pair of monozygotic and a pair of dizygotic twins with a patchy and linear hypopigmentation and autism were reported by Zappella [11]. He found that, in the family of the dizygotic twins, the father had three small depigmented spots and the mother had two depigmented streaks [11]. Our familial case showed only skin involvement without systemic alterations. Regarding the association between HMI and systemic features, Nehal *et al*. suggested that the incidence of associated abnormal features described in previous studies is overstated, and that pigmentary anomalies along the lines of Blaschko are associated with abnormal systemic features less often than previously reported [12]. In their retrospective case series the authors detected that extra-cutaneous abnormal features were present in 16 (30%) of 54 children with aberrant pigmentation along the lines of Blaschko and in particular in only 9 (33%) of 27 with hypomelanosis of Ito. Notably, however, the systemic manifestations can be related to the level of mosaicism in the affected organs. Although we cannot provide evidence of a common origin of the HMI in the father and his daughter in our case report, we postulate that the HMI in the daughter can be due to a rescue of the trisomy 2 zygote. When this is considered, the normal neurologic and systemic development of the young proband allows us to clinically exclude the effects of uniparental disomy (UPD) [13]. It is known that cases of HMI not only show considerable phenotypic variability, but also genotypic variability, with a wide spectrum of chromosomal mosaicisms. A trisomy 2 mosaicism as described in our case report has been reported only once in HMI to the best of our knowledge [14]. The authors described a newborn girl with classic skin findings of HMI with true (postnatal confirmation) trisomy 2 mosaicism. Complete trisomy 2 usually results in first trimester pregnancy losses; trisomy 2 is not fatal only when present in a mosaic style**.** Cases of infants born live with trisomy 2 mosaicism previously described are characterized by intrauterine growth retardation and multiple congenital systemic anomalies [15]. Due to the finding of different chromosomal aberrations, it has been suggested that HMI does not represent a single condition, but rather a non-specific manifestation of chromosomal mosaicism [4]. Our clinical observations suggest that HMI constitutes a general diagnostic definition that can include different sporadic and/or familial clinical phenotypes characterized by the distribution of hypopigmented streaks along the Blaschko lines.

**Table 1:** Reported case of familial hypomelanosis of Ito (HMI)

**Citation**	**Year**	**Authors**	**HMI affected Members**	**Systemic features**	**Karyotype**
[16]	1963	Masumizu	Parents	None	Not performed
[17]	1969	Piñol *et al.*	Mother and her daughter	Myopia, chorioretinal and retinal pigment epithelium atrophy in the right eye	Not performed
[7]	1970	Sacrez *et al.*	Mother and three daughters	Congenital encephalopathy	Normal (peripheral lymphocytes)
					(performed only in one daughter)
[8]	1971	Grosshans *et al.*	Mother and three daughters	Marked psychomotor retardation, strabismus, hypodontia and skeletal dysplasia	Not performed
[18]	1972	Rubin	Two brothers, sister, father and paternal uncle	None	Not performed
[19]	1973	Jelinek *et al.*	Distant and deceased relatives, paternal great aunt	Epileptic seizures and strabismus	Not performed
[9]	1974	Cram and Fukuyama	Child, his mother and his maternal grandfather	Epileptic seizures	Not performed
[20]	1975	Hellgren	Mother, sister and brother	Macrocheilia, iris pigmented spots and hair anomalies	Not performed
[21]	1975	Griffiths and Payne	Mother and father (first cousins)	Ocular hypertelorism, nails and fingers anomalies	Not performed
[22]	1977	Schwartz *et al.*	Nephew and maternal grandmother	Epileptic seizures, retardation, macrocephaly, delayed closure anterior fontanelle, leg length discrepancy, scoliosis and iridial heterochromia	Not performed
[10]	1987	Patrizi *et al.*	Mother and two sibs	Neurological symptoms	Not performed
[23]	1990	Amon *et al.*	Mother and daughter	Ocular symptomatology	Deletion of chromosome 15
					(peripheral lymphocytes and fibroblasts)
[24]	1991	Montagna *et al.*	Mother and two sibs	Mental and cerebellar signs, organic psychosis	Normal (peripheral lymphocytes)
[3]	1992	Vormittag *et al.*	Mother and daughter	Epileptic seizures, ophthalmologic abnormalities, scoliosis and lordosis	Normal (peripheral lymphocytes)
					Tetraploidy in #2 (23%), #5 (11%), #11 and #14 (6%), #18 and #21 (2%) (fibroblasts)
[11]	1993	Zappella	A pair of monozygotic and a pair of dizygotic twins	Autism, delayed psychomotor development and microcephaly	Normal (peripheral lymphocytes)
	2014	Ponti *et al.*	Father and daughter	None	Normal (peripheral lymphocytes)
					Paternal trisomy 2 (fibroblasts)

## Conclusions

Our case remarks the critical role of a careful clinical inspection of all close family members. We therefore suggest the importance of the of apparently innocent and small hypopigmented lesions in order to highlight and demonstrate the presence of a hereditary disorder of pigmentation. Regarding the definition of this peculiar disorder, it is known that the original name incontinentia pigmenti achromians results from the features of the peculiar depigmented changes corresponding to the negative of the morphologic changes characteristic of patients with incontinentia pigmenti [1-3]. However, it is well known that this definition is incorrect, because histologically no incontinent melanin is seen in the dermis, but only a decrease in the number of melanosomes. The most appropriate term hypomelanosis of Ito, pigmentary dysplasia and pigmentary mosaicism are synonyms of the same cutaneous signs. The name Ito syndrome is erroneous as a generic definition of this sporadic condition, and, in our opinion, should be reserved only to familial HMI cases in which the phenotype and the common chromosomal aberrations are documented in at least two first degree-relatives with a systemic involvement of several organs or tissues.

## Consent

Written informed consent was obtained from the patient’s legal guardian(s) (including the patient's father) for publication of this case report and any accompanying images. A copy of the written consent is available for review by the Editor-in-Chief of this journal.

## Abbreviations

HMI: Hypomelanosis of Ito; ISCN: International System for Human Cytogenetic Nomenclature; UPD: uniparental disomy.

## Competing interests

The authors declare that they have no competing interests.

## Authors’ contributions

GP, AP and SS are the principal authors and made major contributions to the writing of the manuscript. AT, CR, VDM, KE, PC, CN, MC and GP analyzed and interpreted the patient data and reviewed the literature. All authors read and approved the final manuscript.
